# Clinical Implications of Having Reduced Mid Forced Expiratory Flow Rates (FEF_25-75_), Independently of FEV1, in Adult Patients with Asthma

**DOI:** 10.1371/journal.pone.0145476

**Published:** 2015-12-30

**Authors:** Craig M. Riley, Sally E. Wenzel, Mario Castro, Serpil C. Erzurum, Kian Fan Chung, Anne M. Fitzpatrick, Benjamin Gaston, Elliot Israel, Wendy C. Moore, Eugene R. Bleecker, William J. Calhoun, Nizar N. Jarjour, William W. Busse, Stephen P. Peters, W. Gerald Teague, Ronald Sorkness, Fernando Holguin

**Affiliations:** 1 Department of Medicine, Division of Internal Medicine, University of Pittsburgh Medical Center, Pittsburgh, Pennsylvania, United States of America; 2 Department of Medicine, Division of Pulmonary, Allergy and Critical Care Medicine, University of Pittsburgh School of Medicine, Pittsburgh, Pennsylvania, United States of America; 3 Department of Medicine, Division of Pulmonary and Critical Care Medicine, Washington University School of Medicine in St. Louis, St. Louis, Missouri, United States of America; 4 Department of Pathobiology, Cleveland Clinic Foundation, Cleveland, Ohio, United States of America; 5 National Heart and Lung Institute, Imperial College London, London, United Kingdom; 6 Department of Pediatrics, Emory University School of Medicine, Atlanta, George, United States of America; 7 Department of Pediatrics, Division of Pediatric Pulmonary Medicine, University Hospitals, Cleveland, Ohio, United States of America; 8 Department of Medicine, Division of Pulmonary and Critical Care Medicine, Brigham and Women’s Hospital, Boston, Massachusetts, United States of America; 9 Department of Medicine, Division of Pulmonary, Critical Care, Allergy and Immunologic Medicine, Wake Forest University School of Medicine, Winston-Salem, North Carolina, United States of America; 10 Department of Medicine, Division of Pulmonary, Critical Care and Sleep Medicine, University of Texas Medical Branch, Galveston, Texas, United States of America; 11 Department of Medicine, Division of Pulmonary and Critical Care Medicine, University of Wisconsin School of Medicine and Public Health, Madison, Wisconsin, United States of America; 12 Department of Medicine, Division of Allergy and Immunology, University of Wisconsin School of Medicine and Public Health, Madison, Wisconsin, United States of America; 13 Department of Pediatrics, Division of Respiratory Medicine, University of Virginia Children’s Hospital, Charlottesville, Virginia, United States of America; 14 School of Pharmacy, University of Wisconsin at Madison, Madison, Wisconsin, United States of America; Research Center Borstel, GERMANY

## Abstract

**Introduction:**

FEF_25-75_ is one of the standard results provided in spirometry reports; however, in adult asthmatics there is limited information on how this physiological measure relates to clinical or biological outcomes independently of the FEV_1_ or the FEV_1_/FVC ratio.

**Purpose:**

To determine the association between Hankinson’s percent-predicted FEF_25-75_ (FEF_25-75_%) levels with changes in healthcare utilization, respiratory symptom frequency, and biomarkers of distal airway inflammation.

**Methods:**

In participants enrolled in the Severe Asthma Research Program 1–2, we compared outcomes across FEF_25-75_% quartiles. Multivariable analyses were done to avoid confounding by demographic characteristics, FEV_1_, and the FEV_1_/FVC ratio. In a sensitivity analysis, we also compared outcomes across participants with FEF_25-75_% below the lower limit of normal (LLN) and FEV_1_/FVC above LLN.

**Results:**

Subjects in the lowest FEF_25-75_% quartile had greater rates of healthcare utilization and higher exhaled nitric oxide and sputum eosinophils. In multivariable analysis, being in the lowest FEF_25-75_% quartile remained significantly associated with nocturnal symptoms (OR 3.0 [95%CI 1.3–6.9]), persistent symptoms (OR 3.3 [95%CI 1–11], ICU admission for asthma (3.7 [1.3–10.8]) and blood eosinophil % (0.18 [0.07, 0.29]). In the sensitivity analysis, those with FEF_25-75_% <LLN had significantly more nocturnal and persistent symptoms, emergency room visits, higher serum eosinophil levels and increased methacholine responsiveness.

**Conclusions:**

After controlling for demographic variables, FEV_1_ and FEV_1_/FVC, a reduced FEF_25-75_% is independently associated with previous ICU admission, persistent symptoms, nocturnal symptoms, blood eosinophilia and bronchial hyperreactivity. This suggests that in some asthmatics, a reduced FEF_25-75_% is an independent biomarker for more severe asthma.

## Introduction

Both the diagnosis of asthma and the monitoring of disease severity rely on the use of pulmonary function testing (PFT) data. Though the forced expiratory volume in one second (FEV_1_) and the FEV_1_ to forced vital capacity (FVC) ratio are the most commonly used spirometric measurements to identify both the presence and degree of airflow obstruction, other values such as the forced expiratory flow between 25% and 75% of the FVC (FEF_25-75_) are also commonly reported [1]. Although variation in the FEF_25-75_ was previously thought to reflect changes exclusively in the small airways (< 2mm) [2–4], subsequent studies using other physiological or radiological parameters have shown that changes in FEF possibly reflect distal airflows that involve airways that have greater diameters. More importantly, there is limited information about its clinical usefulness, including the fact that current guidelines such as those from the Global Initiative for Asthma (GINA) and the Expert Panel Report 3 of the National Heart, Lung and Blood Institute do not provide specific recommendations for the use of FEF_25-75_ in the evaluation or management of asthma [5,6]. In heterogeneous populations, FEF_25-75_ is seldom discordant from FEV_1_ and FEV_1_/FVC [7]. However, reduced FEF_25-75_ in children with asthma has been shown to be associated with increased asthma severity, need for systemic steroid use and more frequent exacerbations in the setting of normal FEV_1_. It is not known whether these results are also applicable to adult asthmatics [8].

To answer this question, we sought to determine whether the percent predicted FEF_25-75_ (FEF_25-75_%) is associated with clinical asthma outcomes among participants of the Severe Asthma Research Program (SARP). We hypothesized that having a reduced FEF_25-75_% would be associated with increased asthma morbidity independent of and beyond the severity implied by more traditional markers like FEV_1_%. We further hypothesized that FEF_25-75_% would be associated with biomarkers linked to more distal airway inflammation.

## Methods

The study population consisted of participants ages 18 or older from the multi-center SARP study who met criteria for asthma and also had FEF measurements. Asthma diagnosis was based on having either a 12% increase in FEV_1_ after short acting bronchodilator or a 20% drop in FEV_1_ after inhalation of methacholine (PC_20_ 25 mg/ml). The SARP 1–2 study has been previously described in detail [9]. Briefly, the study population consisted of subjects recruited at SARP participating academic centers through the use of local advertisement and from their clinics who met eligibility criteria, including being a current nonsmoker with asthma and having less than 5 pack-years of tobacco use. Study participants were classified as having either severe or not severe asthma. According to the American Thoracic Society (ATS) definition, severe asthma was defined as: at least 1 major criteria: a) Use of high-dose inhaled steroids for > 50% of the preceding year or b) continuous or near-continuous oral steroids); and at least 2 minor criteria: a) daily controller medication in addition to inhaled steroids, b) beta agonist required daily or near-daily, c) persistent airway obstruction, d) one or more urgent care visits for asthma per year, e) 3 or more oral corticosteroid bursts/year, f) clinical deterioration with reduction in oral steroid dose, and g) near-fatal asthma event in the past. Non-severe asthmatics included those with moderate (pre bronchodilator FEV_1_ < 80% with or without use of inhaled corticosteroids (CS)) or mild (FEV_1_ ≥ 80% with or without use of inhaled CS) asthma.

### Clinical Data

After signing informed consent, study participants provided demographic information, smoking history, past medical history and frequency of respiratory symptoms in the 3 months preceding enrollment, including cough, sputum production, chest tightness, nighttime asthma symptoms, wheezing, and shortness of breath. Subjects also completed the Juniper Asthma Quality of Life Questionnaire (AQLQ). As this specific project was performed as secondary analysis of the above data following de-identification, no further patient consent or institutional review board approval was required.

### Allergy Skin Test

All participants underwent allergy skin testing for tree mix, grass mix, ragweed, weed mix, dogs, cats, molds, dust mites, and cockroach. To control for validity, diluting fluid and histamine were respectively used as negative and positive controls. Presence of atopy was defined as having at least one skin test reaction of ≥3 mm and greater than the saline control.

### Lung Function testing

Spirometry was done following ATS guidelines[10]. Post bronchodilator FEV_1_ was recorded as the maximum bronchodilator change between 4 and 8 puffs of albuterol. Patients with a baseline FEV_1_ > 50% and FEV_1_ ≥ 1.5L underwent methacholine challenge, following a 7-dose algorithm of incremental doses from 0.078mg/ml to maximum of 25 mg/ml. A provocation concentration (PC20) of <16 mg/ml was considered positive. This high value was chosen because of the high and prolonged steroid doses in the population. Because of FEV_1_ criteria, methacholine challenges were only done to a subset of the subjects. FEF_25-75_% was calculated using Hankinson’s regression models for each sex and by race [11]. Exhaled nitric oxide (eNO) was measured online following ATS and ERS standards [12].

## Statistical Analysis

The FEF_25-75_% distribution was divided into quartiles to determine how the variability in this measure associates with changes in healthcare utilization, frequency of respiratory symptoms and biomarkers of distal airway inflammation. Healthcare utilization included emergency department (ED) visits, hospitalizations and intensive care unit (ICU) admissions for asthma. The frequency of respiratory symptoms was defined as a binary variable for having or not having symptoms at least twice or more per week. Continuous parametric and non-parametric data were respectively compared across FEF_25-75_% quartiles using one way ANOVA or Kruskal Wallis tests with Bonferroni pairwise comparisons. The Chi2 test was used to compare the distribution of categories.

To evaluate for possible confounders, we performed logistic regression analysis that included the following models: a) univariable, b) adjusted for demographic factors (age, gender, BMI, race), and c) full model adjusted for demographic variables + FEV_1_% predicted and the FEV_1_/FVC ratio. The covariable model selection was based a priori and whether their p value was < 0.1 or the model’s estimate changed by ≥ 10%. Given the high degree of correlation between FEF and FEV_1_ or the FEV_1_/FVC ratio, all models were evaluated for collinearity using the variance inflation factor (VIF). Due to the fact that in severe asthmatics, FVC accounts for most of the FEV_1_ reversal after bronchodilation due to air trapping [13], a separate multivariate analysis was run with adjustment for FVC rather than FEV_1_ (S3 Table); both could not be adjusted for in the same model due to collinearity. We also performed a sensitivity analysis to determine the study outcomes of discordant subjects with low FEF_25-75_% but no airway obstruction, defined as having FEF_25-75_ <LLN with FEV_1_/FVC > the lower limit of normal (LLN). All statistics were done using Stata 13.0 (College Park, Tx).

## Results

The characteristics of the study population are shown in Table 1, which consisted of 829 participants, of whom the majority was Caucasian, female and overweight. With decreasing FEF_25-75_% quartiles, there was a greater proportion of African American race, larger BMI, longer duration of asthma and a higher proportion of moderate to severe asthmatics. There was also a strong association between FEF_25-75_% quartiles with increased medication use health care utilization, eNO, IgE and sputum eosinophil proportions. Similarly, participants in the lower quartile distribution were more likely to have required an oral corticosteroid burst, to be seen in the emergency room or to be hospitalized for asthma. Those in the lowest FEF_25-75_% quartile had the highest likelihood of having been previously intubated for asthma. With progressive FEF_25-75_% decline, there was also a significant reduction in FVC, FEV_1_ and the FEV_1_/FVC ratio as shown in Table 2. Adjustment for FVC rather than FEV_1_ did not significantly change the results of the multivariate analysis (S3 Table).

**Table 1:** Characteristics of the study population by the FEF_25-75_% quartile distribution.

		Q1	Q2	Q3	Q4	P-value
	Overall	88 (74–146)	64 (56–74)	46 (37–55)	27 (9–37)	
	N = 829	N = 207	N = 207	N = 208	N = 207	
Demographics						
Age	36.8 (25.5, 46.6)	32.6 (22.2, 38.9)	35.3 (25.6, 42.0)	37.6 (26.7, 47.7)	41.7 (32.2, 49.6)	P<0.0001
Sex—Female	65.1%	67.1%	70.5%	64.4%	58.4%	P = .07
Sex—Male	34.9%	32.9%	29.5%	35.6%	41.6%	
Race—White	64.9%	74.4%	69.6%	61.1%	54.6%	P<0.0001
Race—Black	27.4%	15.0%	25.1%	31.7%	37.7%	
Race—Other	7.7%	10.6%	5.3%	7.2%	7.7%	
BMI	29.9 (23.9, 34.2)	28.0 (22.5, 32.5)	29.6 (23.8, 33.0)	30.8 (24.6, 35.0)	31.3 (25.1, 35.9)	P<0.0001
Duration of asthma (yrs)	21.8 (11.8, 30.6)	17.9 (8.5, 23.8)	19.9 (10.6, 27.1)	23.0 (12.8, 32.1)	26.3 (15.6, 35.9)	P<0.0001
Ever smoked	20.7%	18.0%	20.3%	21.2%	23.2%	P = 0.6
Biomarkers						
Exhaled NO (ppb)	38.0 (15.6, 48.1)	31.1 (13.5, 36.6)	37.1 (14.1, 49.7)	40.1 (17.8, 52.2)	43.8 (17.0, 49.3)	P<0.01*
IgE (IU/mL blood)	324 (54, 334)	197 (42, 226)	316 (52, 355)	401 (64, 362)	384 (56, 385)	P<0.05*
Eosinophil % (blood)	3.93 (2, 5)	3.40 (1.55, 5)	3.95 (2, 5)	3.66 (2, 5)	4.72 (2. 6)	P<0.01**
Sputum eosinophil %	4.89 (0.2, 4.1)	3.22 (0.001, 1.9)	3.08 (0.2, 2.8)	3.66 (0.3, 4.0)	9.59 (0.6, 8.6)	P<0.001**
PC20	1.15 (0.42, 3.89)	2.25 (0.89, 6.48)	1.28 (0.53, 3.99)	0.84 (0.27, 2.42)	0.46 (0.19, 1.44)	P<0.0001*
Outcomes						
ER for breathing ever	67.9%	61.8%	58.5%	68.1%	83.1%	P<0.001
Spent night in hospital ever	42.4%	33.3%	30.4%	38.8%	67.2%	P<0.001
ICU ever	15.9%	6.4%	7.8%	14.7%	34.8%	P<0.001
Intubated ever	8.3%	4.8%	5.8%	4.8%	18.0%	P<0.001

Footnote: Continuous variables are shown as median (Inter quartile range). Categorical variables shown as percentage of total. Q: quartile, BMI: body mass index, NO: nitric oxide, WBC: white blood cell, PC20: provocation challenge, ER: emergency room, ICU: intensive care unit.

* P value calculated from log transformed data

** P value calculated from Kruskal-Wallis test

**Table 2:** Lung function parameters by the FEF25-75% quartile distribution.

	Overall	Q1	Q2	Q3	Q4
	N = 829	N = 207	N = 207	N = 208	N = 207
FEV1 (% predicted)	78.6 (66, 92)	97.2 (90, 104)	87.0 (80, 94)	74.5 (67, 81)	55.9 (46, 66)
FVC (% predicted)	89.4 (79, 101)	97.6 (89, 106)	94.5 (84, 103)	88.1 (79, 97)	77.4 (64, 89)
FEV1/FVC	0.72 (0.65, 0.80)	0.83 (0.80, 0.87)	0.76 (0.73, 0.80)	0.70 (0.66, 0.74)	0.59 (0.53, 0.65)
FEF25-75 (% predicted)	57.0 (37.2, 73.8)	92.5 (80.1, 101.1)	64.0 (59.0, 68.5)	46.0 (41.8, 50.8)	25.6 (20.4, 32.2)

Footnote: Values shown as median (Inter quartile range). Q: quartile, FEV1: forced expiratory volume in one second, FVC: forced vital capacity, FEF25-75: forced expiratory flow between 25% and 75% of FVC.

### Multivariable analyses

#### Respiratory symptoms

**Fig 1:**
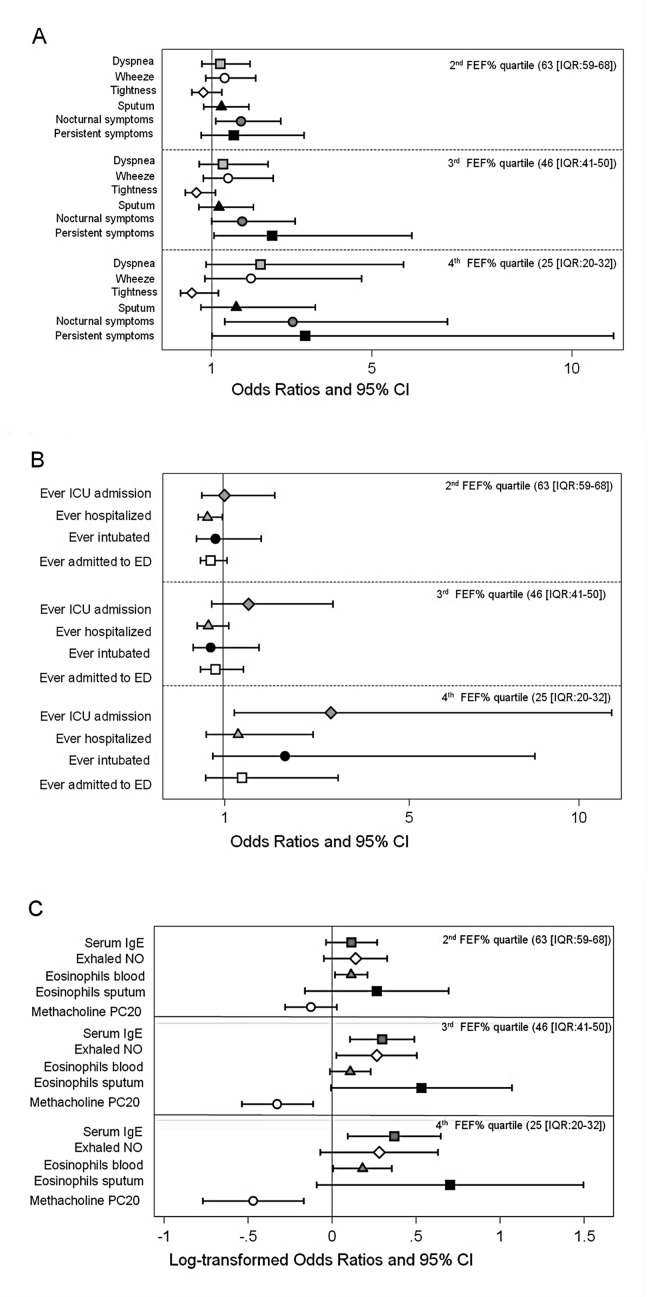
Adjusted odds ratios and beta coefficients of respiratory symptom frequency (a), healthcare utilization (b), biomarkers and bronchial hyperresponsiveness (c). Footnote: Multivariable logistic and regression models adjusted for: for age, sex, body mass index, duration of asthma, history of smoking, FEV1 and FEV1/FVC. Error bars represent 95% confidence intervals.

In the univariable analysis (S1 Table), compared to highest referent FEF_25-75_% quartile (median 92.5 (IQR [80.1, 101.1]) category, the odds of having persistent respiratory symptoms increased in association with lower FEF_25-75_% quartiles and remained largely significant after adjustment for age, BMI, sex, duration of asthma and ever smoking; however, after adjustment for FEV_1_ percent predicted and the FEV_1_/FVC ratio, only nocturnal symptoms and persistent symptoms remained significantly associated with the lowest FEF_25-75_% quartile (median 25.6 [IQR 20.4, 32.2]) as shown in Table 3 and Fig 1A.

**Table 3:** Multivariable analysis of respiratory symptoms, healthcare utilization and biomarkers by FEF_25-75_% quartile distribution.

**Symptoms**	**Wheeze**	**SOB**	**Nocturnal Sx**	**Sputum production**	**Chest tightness**	**Persistent Sx**
Reference (FEF Q1)	1.00	1.00	1.00	1.00	1.00	1.00
FEF Q2	1.32 (0.83, 2.08)	1.21 (0.75, 1.95)	**1.72 (1.09, 2.72)**	1.23 (0.79, 1.91)	0.78 (0.49, 1.24)	1.54 (0.72, 3.30)
FEF Q3	1.40 (0.78, 2.53)	1.27 (0.67, 2.40)	1.75 (0.99, 3.08)	1.17 (0.67, 2.03)	0.60 (0.33, 1.09)	**2.50 (1.05, 5.99)**
FEF Q4	1.97 (0.82, 4.73)	2.21 (0.84, 5.79)	**3.01 (1.32, 6.89)**	1.6 (0.72, 3.58)	0.49 (0.21, 1.15)	**3.32 (1.00, 11.03)**
**Healthcare Usage**	**ER ever**	**Spent night hosp ever**	**ICU ever**	**Ever intubated**		
Reference (FEF Q1)	1.00	1.00	1.00	1.00		
FEF Q2	0.68 (0.42, 1.10)	**0.60 (0.37, 0.98)**	1.04 (0.47, 2.31)	0.91 (0.37, 2.27)		
FEF Q3	0.81 (0.43, 1.52)	0.63 (0.34, 1.14)	1.64 (0.71, 3.78)	0.55 (0.19, 1.62)		
FEF Q4	1.48 (0.56, 3.91)	1.38 (0.58, 3.29)	**3.73 (1.28, 10.83)**	1.63 (0.45, 5.91)		
**Biomarkers**	**eNO**	**IgE**	**Blood Eos**	**Sputum Eos**	**PC20**	
Reference (FEF Q1)	0	0	0	0	0	
FEF Q2	3.37 (-5.05, 11.78)	133.94 (-42.11, 309.99)	**0.07 (0.01, 0.13)**	-0.90 (-4.07, 2.27)	**-1.53 (-2.66, -0.39)**	
FEF Q3	4.85 (-5.84, 15.56)	**255.44 (34.19, 476.69)**	0.07 (-0.01, 0.14)	-0.82 (-4.81, 3.17)	**-2.86 (-4.43, -1.29)**	
FEF Q4	3.38 (-12.28, 19.03)	281.21 (-37.73, 600.14)	**0.18 (0.07, 0.29)**	3.75 (-2.07, 9.57)	***-*3.12 (-5.34, -0.90)**	

Footnote: Multivariable logistic (odds ratio) and linear regression (beta coefficient) models were adjusted for age, sex, body mass index, duration of asthma, history of smoking, FEV1 and FEV1/FVC. Q: quartile, SOB: shortness of breath, Sx: symptoms, ER: emergency room, ICU: intensive care unit, eNO: exhaled nitric oxide, Eos: eosinophils, PC20: provocation challenge, FEF quartiles: Q1: 88 (74–146), Q2: 64 (56–74), Q3: 46 (37–55), Q4: 27 (9–37)

#### Healthcare utilization

In the univariable analysis, compared to the referent category, the odds of ever requiring hospitalizations, ED visits or ICU care increased in relation to lower FEF_25-75_% quartiles (S1 Table). After adjusting for demographics, these associations were attenuated but remained significant. After adjusting for FEV_1_% and the FEV_1_/FVC ratio, only those in the lowest FEF_25-75_% quartile had a significantly increased odds ratio (OR) of ever having an ICU admission for asthma (Table 3, Fig 1B).

#### Biomarkers of allergic, airway inflammation and bronchial hyperactivity

In the univariable analysis, lower FEF_25-75_% quartiles were significantly associated with log-transformed eNO, IgE, PC20, blood and sputum eosinophils (S1 Table). After adjustment for demographics, these remained significant with the exception of blood eosinophilia. In the fully adjusted model, the 3^rd^ FEF_25-75_% quartile was associated with IgE, eNO, PC20, and marginally with sputum eosinophils. In the lowest quartile, only blood eosinophils and PC20 remained significant (Table 3 and Fig 1C), though a trend towards increasing IgE, eNO and sputum eosinophilia remained.

#### Sensitivity analysis on patients with discordant FEF_25-75_ and FEV1/FVC

Compared to asthmatics with an FEF_25-75_ >LLN and FEV_1_/FVC >LLN, those with a reduced FEF_25-75_ <LLN but FEV_1_/FVC >LLN, had significantly increased nocturnal symptoms and persistent symptoms. They were also more likely to visit the emergency room, to have serum eosinophilia and bronchial hyperreactivity (S2 Table).

## Discussion

In this cross sectional study of SARP participants, reduced FEF_25-75_% was associated with increased frequency of respiratory symptoms, greater healthcare utilization and higher levels of biomarkers of distal airway inflammation. Although many of these associations were attenuated by adjusting for FEV_1_% and the FEV_1_/FVC ratio, having a reduced FEF_25-75_% was independently related to more frequent nocturnal and persistent symptoms, ICU admission for asthma, higher eNO, greater bronchial hyperresponsiveness and higher sputum eosinophil percentage. Further, among patients with an FEV_1_/FVC ratio above the lower limit of normal, having an FEF_25-75_ below the LLN was associated with increased symptom burden, healthcare utilization, serum eosinophilia and bronchial hyperreactivity. Together, these results show for the first time in adults with asthma, that having a low FEF_25-75_% identifies a group of patients with higher morbidity and elevated biomarkers of distal airway inflammation.

According to the European Respiratory Society (ERS) and the American Thoracic Society (ATS) task force for standardization of lung function testing, the FEF_25-75_ is defined as the mean forced expiratory flow between the 25% and 75% of the FVC [14,15], which some have interpreted as a quantitative measure of small airways (<2mm) obstruction [4]. Indeed, since the 1970s FEF_25-75_% rates were proposed to be a marker of small airway obstruction and a more sensitive way to detect early stages of obstructive airway disease. However, others have argued that FEF_25-75_% is highly variable and neither sufficiently sensitive nor specific to diagnose obstructive lung disease [16]. Moreover, FEF_25-75_% has not been shown to correlate with other physiologic or histologic measures of distal lung inflammation [16]. Using computed tomography airway morphometric analysis, FEF_25-75_% has been shown to be moderately and inversely correlated with the bronchial wall area (WA) and WA corrected for body surface area, though not exclusively in the small airways [17]. Although it is possible that FEF_25-75_% predominantly reflects flows derived from more distal airways, there is insufficient data to support the concept that variability in this measure is specific to small airway changes. Despite these limitations, FEF_25-75_% continues to be part of the standard spirometry report. More importantly, there are no recommendations as to how reductions in this measure should be taken into consideration for asthma treatment or for risk stratification.

Unlike Quanjer et al[7] who have argued that FEF_25-75_% does not aid clinical decision making, our results support a different conclusion. Although we also found the percentage of discordant cases (low FEF25-75% with a normal FEV1/FVC) is relatively small, FEF was independently associated with several clinical, inflammatory and healthcare outcomes. Further, the study by Quanjer et al was unable to examine these associations, as their study did not include any non-physiological clinical outcomes.

In children, a low FEF_25-75_% has been associated with greater odds for systemic steroids and ED visits, despite having a normal FEV_1_ [18]. Similarly, our study found that even when controlling for FEV_1_ and the FEV_1_/FVC ratio, asthmatics with lower FEF_25-75_% have greater odds of having been admitted to the ICU and to have persistent respiratory symptoms. In addition, we have shown that lower FEF_25-75_% is independently associated with eNO. As shown by Dweik et al[19], eNO is associated with increased asthma morbidity when levels are above 35 ppb. When taken into consideration with increased persistent and nocturnal symptoms, we speculate that FEF_25-75_% is indeed related to more distal airway inflammation either not fully captured or not yet evident by FEV_1_; however, further work must be done to evaluate this hypothesis. If proven in prospective investigation, the clinical implications would include both prognosis and identification of an at-risk asthma population for more intensive therapy.

There are significant limitations that need to be considered when evaluating the results from this study. First, given the cross-sectional nature of this study, no causal implications can be made between FEF_25-75_% and asthma severity. This question will need to be answered in the ongoing longitudinal SARP 3 study. Also, reliance on questionnaire responses may contribute to recall bias, though this should be a non-differential bias with regards to the FEF_25-75_% distributions. Additionally, our results may lack external validity, as the SARP study population is enriched with a higher proportion of participants with severe asthma and is not representative of the general adult asthma population. Though this limits the generalization of these findings, it should be noted that SARP still represents a broader spectrum of asthma than most studies, which may help with distinguishing effects.

## Conclusions

Independent of FEV_1_ and FEV_1_/FVC, FEF_25-75_% predicted identifies a population of adult asthmatics with more severe symptoms, greater health care utilization and elevated biomarkers of distal airway inflammation. Further research is needed to determine if this information can be used clinically to guide treatment decisions or for prognostic evaluation; these questions are currently undergoing longitudinal analysis within SARP 3.

## Supporting Information

S1 Table Symptoms, Health Care Utilization and Biomarkers across FEF Quartiles unadjusted and adjusted for demographics. Footnote: Multivariable logistic (odds ratio) and linear regression (beta coefficient) models were adjusted for age, sex, body mass index, duration of asthma, history of smoking, FEV1 and FEV1/FVC. Bolded values represent p-values < 0.05. *Multivariate analysis odds ratios and 95% confidence intervals. **Linear regression beta coefficients and 95% confidence intervals. Q: quartile, SOB: shortness of breath, Sx: symptoms, ER: emergency room, ICU: intensive care unit, eNO: exhaled nitric oxide, Eos: eosinophils, WBCs: white blood cells, PC20: provocation challenge, FEF quartiles: Q1: 88 (74–146), Q2: 64 (56–74), Q3: 46 (37–55), Q4: 27 (9–37).
(DOC)

S2 Table Sensitivity Analysis Comparing Decreased FEF_25-75_% with Normal FEV_1_/FVC against Normal Spirometry. Footnote: Reference: FEV_1_/FVC >LLN and FEF_25-75_ >LLN. Low/Normal: FEF_25-75_ <LLN and FEV_1_/FVC >LLN. Bolded values are statistically significant to p < 0.05. Values shown represent multivariate analysis odds ratios (*) or linear regression beta coefficients (**). SOB: shortness of breath, Sx: symptoms, ratio: FEV_1_/FVC, FEF: FEF_25-75_%, BMI: body mass index, ER: emergency room, Hosp: hospital, eNO: exhaled nitric oxide, Eos: eosinophils, WBCs: white blood cells, PC20: methacholine provocation challenge.
(DOC)

S3 Table Multivariable analysis of respiratory symptoms, healthcare utilization and biomarkers by FEF_25-75_% quartile distribution with FVC instead of FEV_1_. Footnote: Multivariable logistic (odds ratio) and linear regression (beta coefficient) models were adjusted for age, sex, body mass index, duration of asthma, history of smoking, FVC and FEV1/FVC. Q: quartile, SOB: shortness of breath, Sx: symptoms, ER: emergency room, ICU: intensive care unit, eNO: exhaled nitric oxide, Eos: eosinophils, PC20: provocation challenge, FEF quartiles: Q1: 88 (74–146), Q2: 64 (56–74), Q3: 46 (37–55), Q4: 27 (9–37).
(DOC)

S4 Table Demographic and lung function characteristics of non-obstructed patients with normal vs low FEF_25-75_. Footnote: Continuous variables are shown as median (Inter quartile range). Categorical variables shown as percentage of total. BMI: body mass index, FEV1: forced expiratory volume in one second, FVC: forced vital capacity, FEF25-75: forced expiratory flow between 25% and 75% of FVC. P-values shown calculated by chi-squared for categorical variables and t-test for continuous variables.
(DOC)
