# Supplementary material for: Clinical Implications of Having Reduced Mid Forced Expiratory Flow Rates (FEF25-75), Independently of FEV1, in Adult Patients with Asthma
Source: PLoS One. 2015 Dec 30;10(12):e0145476. doi: 10.1371/journal.pone.0145476 (PMC4696666; doi:10.1371/journal.pone.0145476)
Supplement: S1 Table — Footnote: Multivariable logistic (odds ratio) and linear regression (beta coefficient) models were adjusted for age, sex, body mass index, duration of asthma, history of smoking, FEV1 and FEV1/FVC. Bolded values represent p-values < 0.05. *Multivariate analysis odds ratios and 95% confidence intervals. **Linear regression beta coefficients and 95% confidence intervals. Q: quartile, SOB: shortness of breath, Sx: symptoms, ER: emergency room, ICU: intensive care unit, eNO: exhaled nitric oxide, Eos: eosinophils, WBCs: white blood cells, PC20: provocation challenge, FEF quartiles: Q1: 88 (74–146), Q2: 64 (56–74), Q3: 46 (37–55), Q4: 27 (9–37). (DOC) [file pone.0145476.s001.doc]

Supplemental Table 1.

| **Symptoms** | **Wheeze*** | | **SOB*** | | **Nocturnal Sx*** | | **Sputum production*** | |
| --- | --- | --- | --- | --- | --- | --- | --- | --- |
|  | Model 1 | Model 2 | Model 1 | Model 2 | Model 1 | Model 2 | Model 1 | Model 2 |
| Reference (FEF Q1) | 1 | 1 | 1 | 1 | 1 | 1 | 1 | 1 |
| FEF Q2 | **1.62 (1.10, 2.40)** | **1.58 (1.06, 2.36)** | 1.46 (0.97, 2.18) | 1.35 (0.89, 2.05) | **1.78 (1.19, 2.66)** | **1.74 (1.16, 2.62)** | 1.37 (0.93, 2.01) | 1.31 (0.88, 1.94) |
| FEF Q3 | **2.17 (1.46, 3.21)** | **2.09 (1.38, 3.15)** | **1.80 (1.20, 2.72)** | **1.63 (1.06, 2.51)** | **1.98 (1.33, 2.96)** | **1.90 (1.26, 2.86)** | 1.35 (0.92, 1.99) | 1.25 (0.84, 1.87) |
| FEF Q4 | **4.00 (2.63, 6.09)** | **4.02 (2.56, 6.33)** | **3.81 (2.39, 6.08)** | **3.45 (2.09, 6.67)** | **3.61 (2.41, 5.42)** | **3.49 (2.27, 5.37)** | **2.06 (1.39, 3.05)** | **1.81 (1.19, 2.73)** |
| Age |  | 0.99 (0.98, 1.01) |  | 1.00 (0.99, 1.02) |  | 0.99 (0.98, 1.01) |  | **1.02 (1.01, 1.03)** |
| BMI |  | **1.05 (1.03, 1.07)** |  | **1.04 (1.02, 1.06)** |  | **1.02 (1.01, 1.04)** |  | 1.01 (1.00, 1.03) |
| Sex |  | 0.85 (0.62, 1.16) |  | **1.39 (1.00, 1.93)** |  | 1.14 (0.85, 1.54) |  | 0.98 (0.73, 1.31) |
| Duration of asthma |  | 0.99 (0.98, 1.00) |  | 1.00 (0.98, 1.01) |  | 1.01 (0.99, 1.02) |  | 0.99 (0.98, 1.00) |
| Ever smoked |  | **2.08 (1.41, 3.06)** |  | **2.22 (1.42, 3.45)** |  | **1.65 (1.16, 2.36)** |  | 1.08 (0.76, 1.53) |
|  |  |  |  |  |  |  |  |  |
| **Symptoms** | **Chest tightness*** | | **Persistent Sx*** | |  |  |  |  |
|  | Model 1 | Model 2 | Model 1 | Model 2 |  |  |  |  |
| Reference (FEF Q1) | 1 | 1 | 1 | 1 |  |  |  |  |
| FEF Q2 | 1.20 (0.81, 1.77) | 1.16 (0.77, 1.72) | **3.04 (1.62, 5.70)** | **2.82 (1.48, 5.34)** |  |  |  |  |
| FEF Q3 | 1.31 (0.88, 1.94) | 1.28 (0.85, 1.92) | **12.29 (6.79, 22.24)** | **10.86 (5.92, 19.93)** |  |  |  |  |
| FEF Q4 | **1.80 (1.20, 2.70)** | **1.87 (1.21, 2.88)** | **54.42 (28.89, 102.51)** | **45.82 (23.87, 87.98)** |  |  |  |  |
| Age |  | 0.99 (0.97, 1.00) |  | **1.03 (1.01, 1.05)** |  |  |  |  |
| BMI |  | **1.03 (1.01, 1.05)** |  | **1.04 (1.01, 1.06)** |  |  |  |  |
| Sex |  | 1.31 (0.97, 1.77) |  | **0.5 (0.34, 0.73)** |  |  |  |  |
| Duration of asthma |  | 1.00 (0.99, 1.01) |  | 0.99 (0.98, 1.01) |  |  |  |  |
| Ever smoked |  | **1.61 (1.11, 2.34)** |  | 1.03 (0.67, 1.60) |  |  |  |  |
|  |  |  |  |  |  |  |  |  |
| **Healthcare Useage** | **ER ever*** | | **Spent night hosp ever*** | | **ICU ever*** | | **Ever intubated*** | |
|  | Model 1 | Model 2 | Model 1 | Model 2 | Model 1 | Model 2 | Model 1 | Model 2 |
| Reference (FEF Q1) | 1 | 1 | 1 | 1 | 1 | 1 | 1 | 1 |
| FEF Q2 | 0.87 (0.59, 1.29) | 0.79 (0.52, 1.18) | 0.88 (0.58, 1.33) | 0.78 (0.51, 1.20) | 1.23 (0.58, 2.63) | 1.20 (0.56, 2.59) | 1.21 (0.51, 2.87) | 1.15 (0.48, 2.73) |
| FEF Q3 | 1.32 (0.88, 1.98) | 1.08 (0.70, 1.65) | 1.27 (0.85, 1.90) | 1.00 (0.65, 1.54) | **2.52 (1.27, 4.99)** | **2.29 (1.14, 4.60)** | 1.00 (0.41, 2.46) | 0.88 (0.35, 2.19) |
| FEF Q4 | **3.03 (1.91, 4.80)** | **2.50 (1.52, 4.09)** | **4.09 (2.71, 6.18)** | **3.20 (2.06, 4.99)** | **7.80 (4.15, 14.67)** | **6.94 (3.59, 13.43)** | **4.31 (2.08, 8.93)** | **3.74 (1.74, 8.06)** |
| Age |  | **0.98 (0.96, 0.99)** |  | **0.98 (0.97, 1.00)** |  | 0.99 (0.97, 1.01) |  | 0.99 (0.96, 1.01) |
| BMI |  | **1.05 (1.03, 1.08)** |  | **1.03 (1.01, 1.05)** |  | 1.00 (0.98, 1.03) |  | 1.02 (0.99, 1.05) |
| Sex |  | 1.03 (0.74, 1.43) |  | 0.88 (0.64, 1.20) |  | 0.92 (0.61, 1.38) |  | 1.03 (0.61, 1.75) |
| Duration of asthma |  | **1.04 (1.03, 1.05)** |  | **1.04 (1.03, 1.06)** |  | **1.03 (1.01, 1.05)** |  | 1.02 (1.00, 1.04) |
| Ever smoked |  | 1.34 (0.90, 2.00) |  | 0.91 (0.63, 1.33) |  | 0.70 (0.41, 1.18 |  | 0.75 (0.39, 1.47) |
|  |  |  |  |  |  |  |  |  |
| **Biomarkers** | **eNO**** | | **IgE**** | | **Blood Eos**** | | **Sputum Eos**** | |
|  | Model 1 | Model 2 | Model 1 | Model 2 | Model 1 | Model 2 | Model 1 | Model 2 |
| Reference (FEF Q1) | 0 | 0 | 0 | 0 | 0 | 0 | 0 | 0 |
| FEF Q2 | 5.98 (-1.60, 13.56) | **8.05 (0.57, 15.53)** | 119.04 (-38.59, 276.6) | 120.58 (-37.09, 278.26) | 0.05 (-0.00, 0.10) | 0.05 (-0.00, 0.11) | -0.14 (-2.90, 2.62) | 0.01 (-2.78, 2.80) |
| FEF Q3 | **8.97 (1.29, 16.64)** | **13.06 (5.33, 20.78)** | **204.19 (46.34, 362.03)** | **208.84 (48.58, 369.10)** | 0.03 (-0.02, 0.09) | 0.03 (-0.02, 0.09) | 0.44 (-2.32, 3.21) | 0.78 (-2.06, 3.63) |
| FEF Q4 | **12.70 (5.03, 20.37)** | **18.51 (10.57, 26.45)** | **187.35 (29.29, 345.41)** | **199.38 (33.59, 365.18)** | **0.12 (0.07, 0.17)** | **0.12 (0.06, 0.17)** | **6.38 (3.60, 9.15)** | **6.64 (3.74, 9.55)** |
| Age |  | -0.21 (-0.46, 0.03) |  | **-8.91 (-14.04, -3.79)** |  | 0.00 (-0.00, 0.00) |  | 0.05 (-0.03, 0.14) |
| BMI |  | **-0.69 (-1.01, -0.36)** |  | -0.09 (-7.08, 6.89) |  | -0.00 (-0.00, 0.00) |  | **-0.12 (-0.24, -0.01)** |
| Sex |  | -2.92 (-8.61, 2.76) |  | -58.64 (-175.43, 58.15) |  | -0.02 (-0.06, 0.02) |  | -0.26 (-2.30, 1.79) |
| Duration of asthma |  | -0.22 (-0.45, 0.00) |  | **6.91 (2.14, 11.68)** |  | -0.00 (-0.00, 0.00) |  | -0.05 (-0.13, 0.03) |
| Ever smoked |  | -2.13 (-8.74, 4.47) |  | 133.53 (-4.01, 271.06) |  | -0.01 (-0.06, 0.04) |  | -0.18 (-2.64, 2.27) |
|  |  |  |  |  |  |  |  |  |
| **Biomarkers** | **Sputum WBCs**** | | **PC20**** | |  |  |  |  |
|  | Model 1 | Model 2 | Model 1 | Model 2 |  |  |  |  |
| Reference (FEF Q1) | 0 | 0 | 0 | 0 |  |  |  |  |
| FEF Q2 | 3.59 (-3.37, 10.56) | 3.16 (-3.84, 10.17) | **-1.44 (-2.38, -0.50)** | **-1.59 (-2.52, -0.65)** |  |  | |  |
| FEF Q3 | -3.75 (-10.67, 3.17) | -4.97 (-12.04, 2.09) | **-2.73 (-3.67, -1.79)** | **-3.01 (-3.96, -2.06)** |  |  |  |  |
| FEF Q4 | 5.73 (-1.37, 12.84) | 4.07 (-3.33, 11.46) | **-2.96 (-4.05, -1.86)** | **-3.29 (-4.41, -2.17)** |  |  |  |  |
| Age |  | -0.04 (-0.26, 0.18) |  | **0.07 (0.04, 0.10)** |  |  |  |  |
| BMI |  | -0.05 (-0.35, 0.25) |  | 0.02 (-0.02, 0.07) |  |  |  |  |
| Sex |  | -2.77 (-7.96, 2.43) |  | -0.20 (-0.94, 0.55) |  |  |  |  |
| Duration of asthma |  | **0.23 (0.03, 0.44)** |  | -.02 (-0.05, 0.01) |  |  |  |  |
| Ever smoked |  | -2.21 (-8.30, 3.88) |  | -0.49 (-1.37, 0.40) |  |  |  |  |
