# Supplementary material for: Clinical Implications of Having Reduced Mid Forced Expiratory Flow Rates (FEF25-75), Independently of FEV1, in Adult Patients with Asthma
Source: PLoS One. 2015 Dec 30;10(12):e0145476. doi: 10.1371/journal.pone.0145476 (PMC4696666; doi:10.1371/journal.pone.0145476)
Supplement: S2 Table — Footnote: Reference: FEV1/FVC >LLN and FEF25-75 >LLN. Low/Normal: FEF25-75 LLN. Bolded values are statistically significant to p < 0.05. Values shown represent multivariate analysis odds ratios (*) or linear regression beta coefficients (**). SOB: shortness of breath, Sx: symptoms, ratio: FEV1/FVC, FEF: FEF25-75%, BMI: body mass index, ER: emergency room, Hosp: hospital, eNO: exhaled nitric oxide, Eos: eosinophils, WBCs: white blood cells, PC20: methacholine provocation challenge. (DOC) [file pone.0145476.s002.doc]

Supplemental Table 2.

| **Symptoms** | **Wheeze*** | | **SOB*** | | **Nocturnal Sx*** | | **Sputum production*** | |
| --- | --- | --- | --- | --- | --- | --- | --- | --- |
|  | Model 1 | Model 2 | Model 1 | Model 2 | Model 1 | Model 2 | Model 1 | Model 2 |
| Reference (N=378) | 1 | 1 | 1 | 1 | 1 | 1 | 1 | 1 |
| Low/Normal (N=83) | **1.82 (1.11, 3.00)** | 1.59 (0.95, 2.68) | **2.06 (1.16, 3.65)** | 1.76 (0.96, 3.21) | **2.20 (1.36, 3.957)** | **2.00 (1.21, 3.30)** | 1.02 (0.64, 1.64) | 0.93 (0.56, 1.52) |
| Age |  | 1.01 (1.00, 1.03) |  | 1.02 (1.00, 1.04) |  | **1.02 (1.00, 1.04)** |  | **1.03 (1.01, 1.04)** |
| BMI |  | **1.04 (1.01, 1.06)** |  | **1.05 (1.02, 1.09)** |  | **1.03 (1.00, 1.05)** |  | 1.02 (1.00, 1.04) |
| Sex |  | 0.86 (0.55, 1.34) |  | 1.33 (0.83, 2.13) |  | 1.12 (0.71, 1.78) |  | 0.81 (0.52, 1.25) |
| Duration of asthma |  | 0.99 (0.97, 1.01) |  | 1.00 (0.98, 1.02) |  | 1.00 (0.99, 1.02) |  | 1.00 (0.98, 1.01) |
| Ever smoked |  | 1.48 (0.90, 2.41) |  | **1.84 (1.04, 3.27)** |  | 1.48 (0.92, 2.39) |  | 0.82 (0.51, 1.31) |
|  |  |  |  |  |  |  |  |  |
| **Symptoms** | **Chest tightness*** | | **Persistent Sx*** | |  |  |  |  |
|  | Model 1 | Model 2 | Model 1 | Model 2 |  |  |  |  |
| Reference (N=378) | 1 | 1 | 1 | 1 |  |  |  |  |
| Low/Normal (N=83) | 1.37 (0.83, 2.25) | 1.27 (0.76, 2.13) | **9.82 (5.78, 216.69)** | **11.18 (6.16, 20.27)** |  |  |  |  |
| Age |  | 1.00 (0.98, 1.01) |  | **1.06 (1.04, 1.08)** |  |  |  |  |
| BMI |  | **1.03 (1.01, 1.06)** |  | **1.05 (1.02, 1.08)** |  |  |  |  |
| Sex |  | 1.51 (0.97, 2.35) |  | 0.96 (0.53, 1.74) |  |  |  |  |
| Duration of asthma |  | 1.00 (0.98, 1.02) |  | 1.00 (0.98, 1.02) |  |  |  |  |
| Ever smoked |  | 1.57 (0.95, 2.61) |  | 1.56 (0.87, 2.80) |  |  |  |  |
|  |  |  |  |  |  |  |  |  |
| **Healthcare Useage** | **ER ever*** | | **Spent night in hosp ever*** | | **ICU ever*** | | **Ever intubated*** | |
|  | Model 1 | Model 2 | Model 1 | Model 2 | Model 1 | Model 2 | Model 1 | Model 2 |
| Reference (N=378) | 1 | 1 | 1 | 1 | 1 | 1 | 1 | 1 |
| Low/Normal (N=83) | **2.10 (1.021, 23.64)** | **1.85 (1.03, 3.30)** | **1.84 (1.13, 2.98)** | 1.63 (0.98, 2.72) | **2.15 (1.04, 4.43)** | 1.82 (0.86, 3.89) | 1.26 (0.49, 3.21) | 0.96 (0.36, 2.54) |
| Age |  | 0.99 (0.97, 1.01) |  | 1.00 (0.98, 1.02) |  | 0.99 (0.95, 1.02) |  | 0.97 (0.93, 1.01) |
| BMI |  | **1.04 (1.01, 1.06)** |  | 1.02 (0.99, 1.04) |  | 1.03 (0.99, 1.06) |  | **1.05 (1.01, 1.09)** |
| Sex |  | 1.31 (0.82, 2.11) |  | 1.26 (0.78, 2.05) |  | 1.75 (0.75, 4.10) |  | 1.85 (0.66, 5.22) |
| Duration of asthma |  | **1.04 (1.03, 1.06)** |  | **1.04 (1.02, 1.06)** |  | **1.04 (1.01, 1.07)** |  | **1.05 (1.01, 1.09)** |
| Ever smoked |  | 0.90 (0.54, 1.50) |  | 0.90 (0.54, 1.51) |  | 2.03 (0.97, 4.24) |  | 1.75 (0.72, 4.26) |
|  |  |  |  |  |  |  |  |  |
| **Biomarkers** | **eNO**** | | **IgE**** | | **Blood Eos**** | | **Sputum Eos **** | |
|  | Model 1 | Model 2 | Model 1 | Model 2 | Model 1 | Model 2 | Model 1 | Model 2 |
| Reference (N=378) | 0 | 0 | 0 | 0 | 0 | 0 | 1 | 1 |
| Low/Normal (N=83) | 3.85 (-4.73, 12.43) | 7.43 (-1.28, 16.15) | 130.96 (-41.90, 303.82) | 115.00 (-60.44, 290.44) | **0.12 (0.05, 0.19)** | **0.12 (0.05, 0.20)** | 2.39 (-0.36, 35.13) | 2.41 (-0.41, 5.24) |
| Age |  | -0.03 (-0.34, 0.27) |  | -1.23 (-7.30, 4.84) |  | 0.00 (-0.00, 0.00) |  | -0.00 (-0.10, 0.09) |
| BMI |  | **-0.72 (-1.11, -0.33)** |  | -1.33 (-9.33, 6.67) |  | -0.00 (-0.01, 0.00) |  | -0.03 (-0.16, 0.09) |
| Sex |  | -2.49 (-10.69, 5.71) |  | 45.02 (-109.13, 199.16) |  | -0.02 (-0.09, 0.04) |  | 0.40 (-2.13, 2.93) |
| Duration of asthma |  | -0.19 (-0.48, 0.09) |  | **8.68 (2.99, 14.36)** |  | 0.00 (-0.00, 0.00) |  | 0.03 (-0.06, 0.12) |
| Ever smoked |  | -45.16 (-13.87, 3.54) |  | 4.96 (-160.29, 170.21) |  | -0.05 (-0.12, 0.02) |  | -0.25 (-2.96, 2.46) |
|  |  |  |  |  |  |  |  |  |
| **Biomarkers** | **Sputum WBCs**** | | **PC20**** | |  | |  |  |
|  | Model 1 | Model 2 | Model 1 | Model 2 |  |  |  |  |
| Reference (N=378) | 0 | 0 | 0 | 0 |  |  |  |  |
| Low/Normal (N=83) | -3.31 (-12.14, 5.53) | -4.23 (-13.31, 4.85) | **-2.30 (-3.68, -0.93)** | **-2.37 (-3.76, -0.99)** |  |  |  |  |
| Age |  | -0.06 (-0.38, 0.25) |  | 0.04 (-0.01, 0.08) |  |  |  |  |
| BMI |  | 0.02 (-0.40, 0.43) |  | 0.03 (-0.03, 0.09) |  |  |  |  |
| Sex |  | -3.74 (-12.10, 4.61) |  | -1.17 (-2.36, 0.03) |  |  |  |  |
| Duration of asthma |  | 0.18 (-0.11, 0.47) |  | **-0.06 (-0.10, -0.02)** |  |  |  |  |
| Ever smoked |  | -0.21 (-8.89, 8.48) |  | -0.04 (-1.34, 1.27) |  |  |  |  |
