# Supplementary material for: Clinical Implications of Having Reduced Mid Forced Expiratory Flow Rates (FEF25-75), Independently of FEV1, in Adult Patients with Asthma
Source: PLoS One. 2015 Dec 30;10(12):e0145476. doi: 10.1371/journal.pone.0145476 (PMC4696666; doi:10.1371/journal.pone.0145476)
Supplement: S3 Table — Footnote: Multivariable logistic (odds ratio) and linear regression (beta coefficient) models were adjusted for age, sex, body mass index, duration of asthma, history of smoking, FVC and FEV1/FVC. Q: quartile, SOB: shortness of breath, Sx: symptoms, ER: emergency room, ICU: intensive care unit, eNO: exhaled nitric oxide, Eos: eosinophils, PC20: provocation challenge, FEF quartiles: Q1: 88 (74–146), Q2: 64 (56–74), Q3: 46 (37–55), Q4: 27 (9–37). (DOC) [file pone.0145476.s003.doc]

_Supplemental Table 3_.

| **Symptoms** | **Wheeze** | **SOB** | **Nocturnal Sx** | **Sputum production** | **Chest tightness** | **Persistent Sx** |
| --- | --- | --- | --- | --- | --- | --- |
| Reference (FEF Q1) | 1 | 1 | 1 | 1 | 1 | 1 |
| FEF Q2 | 1.34 (0.85, 2.11) | 1.23 (0.76, 1.98) | **1.79 (1.14, 2.82)** | 1.19 (0.77, 1.85) | 0.80 (0.51, 1.25) | 1.69 (0.81, 3.54) |
| FEF Q3 | 1.49 (0.84, 2.64) | 1.28 (0.69, 2.37) | **1.97 (1.14, 3.41)** | 1.12 (0.66, 1.91) | 0.64 (0.36, 1.13) | **3.27 (1.40, 7.61)** |
| FEF Q4 | 2.07 (0.88, 4.83) | 2.16 (0.85, 5.48) | **3.58 (1.61, 7.95)** | 1.52 (0.70, 3.29) | 0.51 (0.22, 1.18) | **5.27 (1.64, 16.92)** |
|  |  |  |  |  |  |  |
| **Healthcare Useage** | **ER ever** | **Spent night hosp ever** | **ICU ever** | **Ever intubated** |  |  |
| Reference (FEF Q1) | 1 | 1 | 1 | 1 |  |  |
| FEF Q2 | 0.69 (0.43, 1.11) | **0.62 (0.38, 0.99)** | 1.09 (0.49, 2.42) | 0.93 (0.37, 2.31) |  |  |
| FEF Q3 | 0.81 (0.44, 1.50) | 0.65 (0.36, 1.16) | 1.80 (0.80, 4.09) | 0.57 (0.20, 1.63) |  |  |
| FEF Q4 | 1.45 (0.56, 3.72) | 1.42 (0.61, 3.28) | **4.26 (1.53, 11.88)** | 1.67 (0.48, 5.74) |  |  |
|  |  |  |  |  |  |  |
| **Biomarkers** | **eNO** | **IgE** | **Blood Eos** | **Sputum Eos** | **PC20** |  |
| Reference (FEF Q1) | 0 | 0 | 0 | 0 | 0 |  |
| FEF Q2 | 3.19 (-5.11, 11.49) | 126.07 (-48.12, 300.27) | **0.07 (0.01, 0.13)** | -0.93 (-4.05, 2.19) | **-1.57 (-2.70, -0.45)** |  |
| FEF Q3 | 4.41 (-5.87, 14.68) | **236.95 (23.90, 450.00)** | 0.07 (-0.00, 0.14) | -0.92 (-4.77, 2.93) | **-2.98 (-4.52, -1.45)** |  |
| FEF Q4 | 2.48 (-12.55, 17.52) | 266.15 (-40.34, 572.63) | **0.18 (0.08, 0.29)** | 3.43 (-2.19, 9.05) | **-3.29 (-5.47, -1.11)** |  |
