# Supplementary material for: Clinical Implications of Having Reduced Mid Forced Expiratory Flow Rates (FEF25-75), Independently of FEV1, in Adult Patients with Asthma
Source: PLoS One. 2015 Dec 30;10(12):e0145476. doi: 10.1371/journal.pone.0145476 (PMC4696666; doi:10.1371/journal.pone.0145476)
Supplement: S4 Table — Footnote: Continuous variables are shown as median (Inter quartile range). Categorical variables shown as percentage of total. BMI: body mass index, FEV1: forced expiratory volume in one second, FVC: forced vital capacity, FEF25-75: forced expiratory flow between 25% and 75% of FVC. P-values shown calculated by chi-squared for categorical variables and t-test for continuous variables. (DOC) [file pone.0145476.s004.doc]

Supplemental Table 4.

|  | **Normal FEF N=378** | **FEF<LLN N=83** | **P-value** |
| --- | --- | --- | --- |
| Demographics |  |  |  |
| Age (years) | 36.19 (24.59, 46.05) | 37.97 (27.72, 47.86) | 0.240 |
| Sex |  |  | 0.036 |
| *Female* | 77.25% | 66.27% |  |
| *Male* | 22.75% | 33.73% |  |
| Race |  |  | 0.055 |
| *White* | 67.46% | 57.83% |  |
| *Black* | 25.66% | 27.71% |  |
| *Other* | 6.88% | 14.46% |  |
| BMI (kg/m^2^) | 29.64 (23.30, 33.90) | 32.96 (27.72, 47.86) | 0.002 |
| Duration of asthma (years) | 19.50 (9.16, 26.91) | 22.65 (11.89, 31.50) | 0.045 |
| Ever smoked | 18.83% | 25.30% | 0.183 |
|  |  |  |  |
| Lung Function |  |  |  |
| FEF25-75, Liters/second | 2.94 (2.22, 3.58) | 1.70 (1.26, 1.97) | 0.000 |
| FEF25-75, %predicted | 77.98 (63.92, 89.33) | 47.17 (37.57, 53.84) | 0.000 |
| FEV1, Liters | 3.02 (2.46, 3.50) | 2.27 (1.83, 2.60) | 0.000 |
| FEV1, %predicted | 91.37 (83.00, 100.00) | 70.06 (62.00, 78.00) | 0.000 |
| FEV1/FVC | 0.81 (0.77, 0.85) | 0.75 (0.73, 0.77) | 0.000 |
